# Whole exome sequencing in the rat

**DOI:** 10.1186/s12864-018-4858-8

**Published:** 2018-06-20

**Authors:** Julie F. Foley, Dhiral P. Phadke, Owen Hardy, Sara Hardy, Victor Miller, Anup Madan, Kellie Howard, Kimberly Kruse, Cara Lord, Sreenivasa Ramaiahgari, Gregory G. Solomon, Ruchir R. Shah, Arun R. Pandiri, Ronald A. Herbert, Robert C. Sills, B. Alex Merrick

**Affiliations:** 1 Biomolecular Screening Branch, National Institute of Environmental Health Sciences, 111 T.W. Alexander Dr. Research Triangle Park, Durham, NC USA; 2 Epigenetics and Stem Cell Biology Laboratory, National Institute of Environmental Health Sciences, Research Triangle Park, Durham, NC USA; 3 Cellular and Molecular Pathology Branch, National Institute of Environmental Health Sciences, Research Triangle Park, Durham, NC USA; 4 Sciome, LLC, Research Triangle Park, Durham, NC USA; 5 Agilent Technologies, Santa Clara, CA USA; 6 Covance Genomics Laboratory, Redmond, WA USA; 7 Adaptive Biotechnologies, Seattle, WA USA

**Keywords:** Whole exome sequencing, Next generation sequencing, C6, FAT7, DSL-6A/C1, NBTII, Sanger, COSMIC

## Abstract

**Background:**

The rat genome was sequenced in 2004 with the aim to improve human health altered by disease and environmental influences through gene discovery and animal model validation. Here, we report development and testing of a probe set for whole exome sequencing (WES) to detect sequence variants in exons and UTRs of the rat genome. Using an in-silico approach, we designed probes targeting the rat exome and compared captured mutations in cancer-related genes from four chemically induced rat tumor cell lines (C6, FAT7, DSL-6A/C1, NBTII) to validated cancer genes in the human database, Catalogue of Somatic Mutations in Cancer (COSMIC) as well as normal rat DNA. Paired, fresh frozen (FF) and formalin-fixed, paraffin-embedded (FFPE) liver tissue from naive rats were sequenced to confirm known dbSNP variants and identify any additional variants.

**Results:**

Informatics analysis of available gene annotation from rat RGSC6.0/rn6 RefSeq and Ensembl transcripts provided 223,636 unique exons representing a total of 26,365 unique genes and untranslated regions. Using this annotation and the Rn6 reference genome, an in-silico probe design generated 826,878 probe sequences of which 94.2% were uniquely aligned to the rat genome without mismatches. Further informatics analysis revealed 25,249 genes (95.8%) covered by at least one probe and 23,603 genes (93.5%) had every exon covered by one or more probes. We report high performance metrics from exome sequencing of our probe set and Sanger validation of annotated, highly relevant, cancer gene mutations as cataloged in the human COSMIC database, in addition to several exonic variants in cancer-related genes.

**Conclusions:**

An in-silico probe set was designed to enrich the rat exome from isolated DNA. The platform was tested on rat tumor cell lines and normal FF and FFPE liver tissue. The method effectively captured target exome regions in the test DNA samples with exceptional sensitivity and specificity to obtain reliable sequencing data representing variants that are likely chemically induced somatic mutations. Genomic discovery conducted by means of high throughput WES queries should benefit investigators in discovering rat genomic variants in disease etiology and in furthering human translational research.

**Electronic supplementary material:**

The online version of this article (10.1186/s12864-018-4858-8) contains supplementary material, which is available to authorized users.

## Background

The laboratory rat is a useful mammalian model for the translation and validation of human gene-function discovery toward understanding the interplay between genetics, environmental influences and disease biology. As an experimental animal in toxicology and safety pharmacology applications, the rat is often the model of choice because of its relatively large size and its biologic relevance to human physiology, disease and histopathology. In the past decade, its research popularity has continued because of molecular sequencing advancements through Next Generation Sequencing (NGS). The rat genome was sequenced in 2004 [1] and refinements such as an RNA-Seq expression atlas [2] and genomic updates continue to improve our understanding [3] of the species. Many rat strains are broadly used in toxicology and pharmacology studies and recent developments in genome editing technologies such as CRISPR/Cas-9, have significantly increased the library of available rat strains as targeted disease models for gene discovery and validation [4, 5].

Exome sequencing provides an efficient method to examine sequence variants in coding regions that are related to disease without sequencing the entire genome [6]. Conventional sequencing methods rely upon hybridization of probes from fragmented DNA that are designed around sequences of exonic regions. Various platforms have adopted different probe types and capture chemistries involving overlapping, tiling or gapped probes. Exon size, GC content, repeat elements, and segmental duplications are factors in probe design affecting exon coverage [7]. A recent report on a rat probe set called TargetEC [8], based on the rat reference genome from assembly Rnor_5 was designed to capture coding exonic regions and conserved non-coding regulatory sequences from 13 vertebrate species. The entire probe set covered a 146.8 Mb genomic region. Two control inbred rat strains (WTC/Kyo, PVG/Seac) and 2 mutant strains (WTC-swh/Kyo, KFRS4/Kyo) with known disease mutations were examined. The WES performance metrics for capture specificity and sensitivity were acceptable with 85–94% reads from exomes after removing duplicates and ~ 79% reads on target with an average target depth ranging from 107 to 125-fold. Sequencing validation was minimal, evaluating only two captured mutations previously identified as responsible for disease phenotypes in mutant rat strains [8]. The large target size of capture probes at 146.8 Mb may limit the utility of this platform due to the sequence depth required for the detection of low frequency or rare sequence variants. Thus, other novel approaches are needed to achieve sufficient performance that may enable evaluation of potentially human-relevant disease causing mutations in the rat genome.

**Fig. 1:**
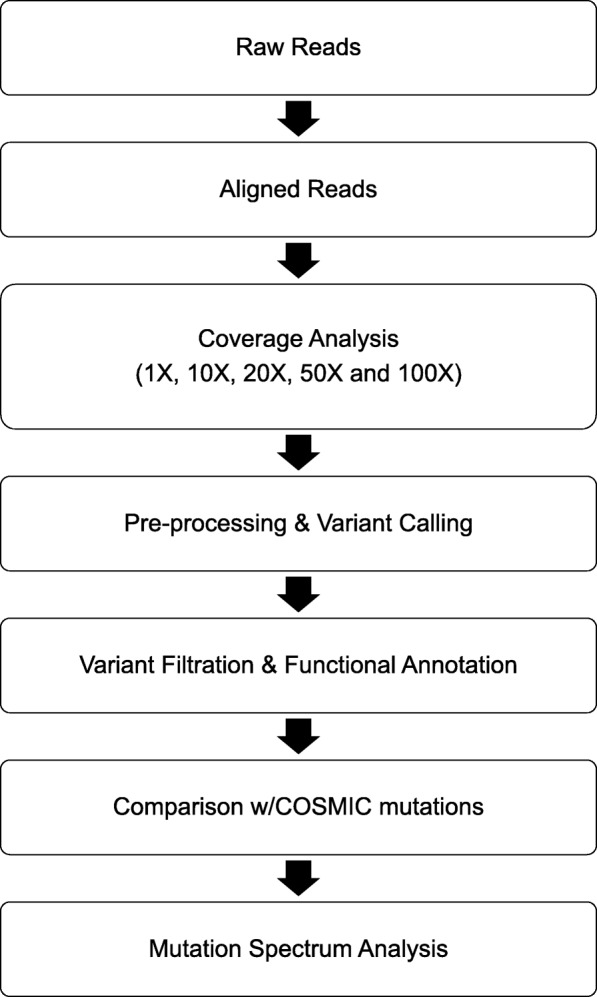
Bioinformatic evaluation pipeline for variant detection. Initially, raw reads were mapped and trimmed followed by targeted coverage analysis, filtration and functional annotation of variant calls. The resulting calls were then compared to validated cancer gene variants in the human COSMIC database. The final analysis involved assessment of the mutational spectrum from the tested samples

Here we describe an in-silico probe design for the evaluation and validation of a WES platform specifically for the rat based on the updated Rnor_6.0 assembly using RefSeq and Ensembl annotations. Tiling probes with a 1 bp overlap were designed, and libraries constructed, using the Agilent SureSelect® XT target enrichment system. Naive, paired FF and FFPE rat liver DNA samples served as control rat exomes and were compared against four chemically induced, rat tumor cell lines (C6, FAT7, DSL-6A/C1 and NBTII) available through American Type Culture Collection (ATCC). Bioinformatic evaluation (Fig. 1) of the FF-FFPE samples revealed cataloged SNPs as well as some not reported in dbSNP (Build 149; November 7, 2016). We were able to affirm in the chemically induced, tumor cell lines, high quality sequence variants associated with common, cancer-related genes annotated in the COSMIC database (v82). Together these data demonstrate WES can be a valuable tool for high throughput sequencing (HTS) in rat models of disease and chemical exposure.

## Methods

### Tissue samples

Samples for evaluating the rat exome-seq platform consisted of DNA extracted from control, non-treated rat liver tissue and chemically induced rat tumor cell lines (American Type Culture Collection, Manassas, VA) (Table 1).

**Table 1:** Rat exome-seq platform study samples

Sample Name	Type	Treatment	Rat Strain
C6	Glioma	N,N-nitroso-methylurea	Wistar
FAT7	Nasal cavity squamous cell carcinoma	Formaldehyde	Fisher-344
DSL-6A/C1	Pancreatic acinar carcinmoma	Azaserine	Lewis
NBTII	Surface epithelial bladder carcinoma	N-butyl-N-(4-hydroxybutyl)nitrosamine	Wistar
FF1	Fresh frozen	Normal liver	Sprague Dawley
FF2	Fresh frozen	Normal liver	Sprague Dawley
FF3	Fresh frozen	Normal liver	Sprague Dawley
FF4	Fresh frozen	Normal liver	Sprague Dawley
FFPE1	Formalin-fixed, paraffin-embedded	Normal liver	Sprague Dawley
FFPE2	Formalin-fixed, paraffin-embedded	Normal liver	Sprague Dawley
FFPE3	Formalin-fixed, paraffin-embedded	Normal liver	Sprague Dawley
FFPE4	Formalin-fixed, paraffin-embedded	Normal liver	Sprague Dawley

Biological replicates of liver from four naive, male, Sprague Dawley retired breeders (CRL:SD, Taconic Biosciences, Hudson, NY), 6–9 months of age, were used as normal controls for the study. Fresh frozen tissue samples served as the “gold standard” for the rat WES evaluation to compare with paired FFPE tissue and the chemically induced, tumor cell lines with documented mutations selected as positive controls for obtaining exonic mutations in cancer-related genes. Experiments were performed according to the guidelines established in the NIH Guide for the Care and Use of Laboratory Animals (National Research Council, 2011). All animals were treated humanely for alleviation of potential suffering, as approved by the National Institute of Environmental Health Sciences Animal Care and Use Committee.

Following euthanasia with CO_2,_ paired FF and FFPE samples were prepared from the left lobe of each animal. A single, representative section (3 mm) was fixed overnight (18–24 h) in 10% neutral-buffered formalin, routinely processed and embedded in paraffin. The remainder of the lobe was cubed (3–5 mm), flash-frozen in liquid nitrogen, and stored at − 80 °C. FFPE blocks were stored at room temperature and sectioned within two months of tissue embedding. All sectioning was conducted under sterile, nuclease-free conditions. Prior to sectioning, the block was trimmed to minimize paraffin surrounding the tissue. A nuclease-free, water pre-soak for 30 s at room temperature prevented tissue chattering. Three to five, 10 μm sections of the FFPE block, dependent upon tissue area were collected in a sterile cryovial and stored at − 20 °C until DNA isolation.

### Cell line samples

To meet the study objective of identifying variants in a newly developed rat exome method, we purposely aimed for toxicology relevant, chemically induced cancer cell lines that contained mutations associated with a cancer phenotype. Four, rat tumor cell lines induced by chemical exposure (C6; glioma, FAT7; nasal squamous cell carcinoma, DSL-6A/C1; pancreatic acinar carcinoma and NBTII; surface epithelial bladder tumor) were used for the testing of the WES platform. The number of cells processed for each respective cell line was C6 (3 × 10^6^), FAT7 (3.1 × 10^6^), DSL-6A/C1 (3.1X10^6^), and NBTII (1.3 × 10^6^). Cell lines were thawed, suspended in 10 mL of media according to the manufacturer’s specifications and spun (Eppendorf 5810R centrifuge, Hauppauge, NY) at 172 x g (1000 rpm) for 5 min. The supernatant was discarded, the cells suspended in 200 μL of 1X PBS and immediately processed for DNA isolation.

### Genomic DNA purification

Qiagen kits (Qiagen, Germantown, MD) were used according to manufacturer’s instructions for genomic DNA isolation from the paired FF (QIAamp Fast DNA Tissue Kit) and FFPE (GeneRead DNA FFPE Kit) liver tissue, and the rat tumor cell lines (Blood and Cell Culture DNA Mini Kit). Sample integrity and yield were assessed by the Nanodrop® (Thermo Fisher Scientific, Madison, WI), the Qubit® Fluorometer (Thermo Fisher Scientific) and the Agilent TapeStation® (Santa Clara, CA). Purified DNA samples were stored at − 20 °C.

### In-silico probe design

RefSeq and Ensembl gene annotations for the rat reference genome from assembly Rnor_6.0 (RGSC, 2014) were downloaded from the UCSC Genome Browser [9]. RefSeq and Ensembl annotations covered protein coding, lncRNAs and miRNAs with no RefSeq overlap and for which exons were removed that were completely contained in other longer exons. Merging the two annotations, the in-silico probe design covered a total of 223,636 exons (26,365 genes) of the reference rat exome. Based on the input targets, 120-base RNA probes were created iteratively by tiling at approximately 1X density (end-to-end) along the sense strand of the reference genome, Rnor_6.0 assembly. Probe filtering was based on the uniqueness criteria of an exon read having a single chromosome location within the Rn6 genome. Low complexity or repetitive probes not meeting the Rn6 genome criteria were removed from the pool. In total, 826,878 probe sequences were combined and used to manufacture a single biotinylated RNA library for target capture.

### Exome enrichment and sequencing

Sequencing libraries were created using the Agilent SureSelect®XT method with on-bead modifications. Genomic DNA (150 ng) was fragmented to approximately 150 bp with a Covaris sonicator (Woburn, MA) and purified with Agencourt AMPure XP beads (Beckman Coulter Genomics, Brea, CA) according to the manufacturer’s specifications. Post-capture library amplifications and quality assessments were also performed according to manufacturer’s specifications. Illumina sequencing on the HiSeq2500® platform (San Diego, CA) was performed by Q^2^ Solutions (Morrisville, NC) at a 75X exome coverage from 2 × 100 bp paired-end reads. Libraries were bar-coded and multiplexed in a pool of 4 samples over 3 lanes. The Illumina Casava® software (v1.8) was used to make base calls. Sequences were output in FASTQ format. Raw data were deposited in GEO (PRJNA434726).

### Variant detection

FASTQ files were subjected to quality control with the FastQC tool (www.bioinformatics.babraham.ac.uk/projects/fastqc/). Read pairs were mapped to the Rn6 genome using the BWA alignment tool (v0.7.12) [10]. Any exon reads whose two ends mapped on different chromosomes were discarded and considered ambiguous as the read did not match the probe filtering criteria of an exon matching to a single chromosome. Duplicates were trimmed from the reads using the MarkDuplicates program from Picard tools (v1.99). Utilities from the BEDTools package were used to obtain the coverage at each target base. Coverage was summarized at the following different levels: 1X, 10X, 20×, 30X and 50X. Variants were called using Genome Analysis Toolkit (GATK; v3.7) [11]. SNVs and INDEL calling was performed with the GATK utility, HaplotypeCaller (https://software.broadinstitute.org/gatk/documentation/tooldocs/current/org_broadinstitute_gatk_tools_walkers_haplotypecaller_HaplotypeCaller.php). Exonic variant filtration was done with GATK, followed by functional annotation of the variants with the SnpEff tool [12]. To determine if there was human disease relevance between our chemically induced tumor mutations and previously identified mutations found in human malignant tumors, we matched the variants in each cell line to validated mutations present in the COSMIC database with the same amino acid substitutions and/or location as that found in our exome sequence data. The mutation spectrum of the final variant set was analyzed with the R package, SomaticSignatures (v3.6; Bioconductor), [13] and compared with different mutational processes that generate unique combinations of mutation types termed mutational signatures in the Catalogues of Somatic Mutations In Cancer (COSMIC) database [14].

### Sequence validation

SNPs from the four chemically induced, rat tumor cell lines detected by WES with medium (non-synonymous missense, nonsense) or high (frameshift mutations and INDELs) functional effects were selected for orthogonal testing by Sanger Sequencing. Variants included sequencing of three well-studied, cancer-related genes (*Tp53, Pik3ca*, and *Ncor1*) along with a subset of 15 additional cancer-related genes (*Nf1, Mki67, Mllt4, Fgfr2, Ctnnb1, Fat1, Clp1, Fat4, Arid1a, Nat1, Nat2, Setd2, Impg1, Nbas,* and *Npat*). Primers were designed from 500 bp flanking nucleotide sequences of the Rnor_6 rat assembly for each sequence variant (Additional file 1: Table S1). Two gene deletions (*Cdkn2a, Cdkn2b*) called by WES were evaluated by qPCR analysis.

### Sanger sequencing

PCR amplification of various regions from the designated cancer-related genes was performed using KAPA Hyper polymerase (Roche Holding AG, Basil, CH) with the primer pairs found in Additional file 2: Table S2. Forward primers were tagged with M13 forward sequences and reverse primers tagged with M13 reverse sequences. The reaction was as follows: after preheating at 95 °C for 5 min, amplification consisted of 30 cycles at 98 °C for 20 s, 65 °C for 15 s and 72 °C for 15 s, and a final extension at 72 °C for 5 min. PCR products were purified using the AMpure® XP (Beckmann Coulter, Brea, CA) and quantified using the NanoDrop®. DNA (10 ng) was sequenced using the BigDye Terminator® cycle sequencing kit (v3.1; Thermo Fisher Scientific). Following purification using the Centri-Sep® Spin Columns (Thermo Fisher Scientific), the nucleotide sequences were determined using an ABI3730XL Genetic Analyzer (Thermo Fisher Scientific). Sequence data was aligned using Sequencher® DNA sequence analysis software (v5.2.4; Gene Codes Corporation, Ann Arbor, MI).

### qPCR analysis for gene (Cdkn2a and Ckdn2b) deletion

DNA study samples from the four rat cell lines along with one normal rat liver control sample and two no template controls were subjected to qPCR analysis using TaqMan® qPCR CNV Assays on the Applied Biosystems 7900HT Fast Real-Time PCR System (Foster City, CA). TaqMan® CNV Master mix was prepared using 1.25 μL of 20× from either one of three custom designed assays specific to *Cdkn2a* or *Cdkn2b* and one of the two copy number reference assays (*APPRKTP* and *APRWFDM*). Reactions were set using 2 μL (5 ng/μL) of DNA samples and 8 μL of CNV master mix (Thermo Fisher Scientific). Each sample was analyzed in duplicate with denaturation at 95 °C for 10 min, followed by 40 cycles at 95 °C for 15 s, and 60 °C for 1 min. Data were analyzed using Applied Biosystems CopyCaller™ Software (v2.1; Thermo Fisher Scientific).

## Results

Rat exome capture probes were designed to capture 71 Mb of the rat genome. An initial experiment was performed to test the capture ability of the probe set on exons and flanking regions from 26,365 rat genes. Enriched DNA fragments were sequenced on an Illumina HiSeq2500® platform from four paired FF and FFPE rat liver samples and four chemically induced, rat tumor cell lines.

### Probe performance

Sequencing was performed at an average of 75-fold depth in exome coverage. The mean ± SEM number of bases sequenced was 19.1 ± 0.202 Gb for the FF liver, 17.3 ± 0.335 Gb for the FFPE liver and 18.2 ± 0.297 Gb for the cell lines with minimal variability among sample types. Liver and cell line sample reads mapped at 99% to the Rn6 reference genome (Table 2).

**Table 2:** Summary statistics for rat WES reads

Sample	Total Reads	Aligned Reads	Aligned Reads (%)	Reads in Target Exons	Reads on Target (%)	Duplicate Reads (%)
C6	171M	170 M	98.9	135 M	79.5	32.3
DSL-6A/C1	179 M	178 M	99.1	141 M	79.1	27.5
FAT7	185 M	183 M	99.1	146 M	79.6	29.6
NBTII	176 M	174 M	99.1	139 M	79.9	27.6
FF1	187 M	185 M	99.2	146 M	79.0	22.7
FF2	181 M	180 M	99.1	141 M	78.4	21.8
FF3	189 M	187 M	99.0	150 M	79.8	24.9
FF4	189 M	187 M	99.0	149 M	79.9	23.0
FFPE1	172 M	159 M	92.0	118 M	74.4	46.9
FFPE2	174 M	165 M	95.0	128 M	77.3	42.7
FFPE3	160 M	134 M	84.0	81 M	60.3	49.1
FFPE4	170 M	150 M	88.2	105 M	70.4	44.1

Read length was 101 bp for all samples

*M* Million

Approximately 80% of the reads aligned to target exons in FF and cell line samples. Target read duplicates were relatively low at a mean of 23% ± 0.6 for FF liver and 29% ± 1.0 for cell lines, but were almost doubled in FFPE samples at 45% ± 1.3.

### Rat exome-Seq performance

**Fig. 2:**
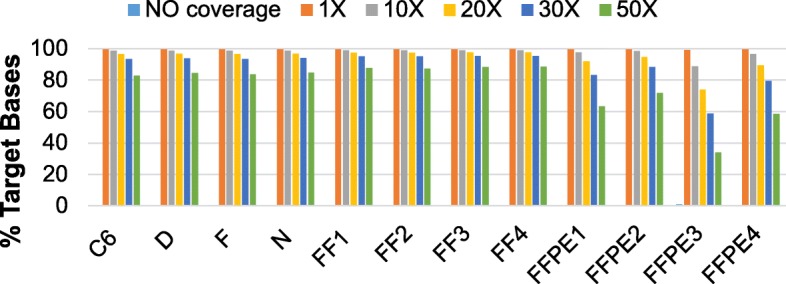
Breadth of reference genome coverage. The percentage of target bases covering the rat Rn6 reference genome is shown at 1X, 10X, 20X, 30X and 50X depth of coverage. Rigourous testing at 50X demonstrated strong coverage of the rat reference genome by the sequenced fragments

Performance characteristics include the number of reads per base for each exon and uniformity for depth of coverage. Minimum depth of coverage is typically 10–20-fold for accurate base calls [15, 16]. We analyzed base pair coverage at 1X, 10X, 20X, 30X and 50X to examine the effects of increasing levels of coverage stringency (Fig. 2). Capture sensitivity was consistent across the three sample types and within the sample groups. At a minimum coverage depth of 10X, ~ 98% of the target bases were sequenced for all samples with the exception of one FFPE sample (FFPE3), which may be related to higher sample fragmentation with formalin fixation [17]. A more stringent depth of coverage cut off at 50X was selected to minimize false positive variant detection; 50X coverage showed acceptable base pair coverage for the FF (88%) and cell line (84%) samples, while we noted the FFPE coverage lowered to ~ 57%. Target regions not covered by any reads were negligible with less than 1% of non-covered bases.

**Fig. 3:**
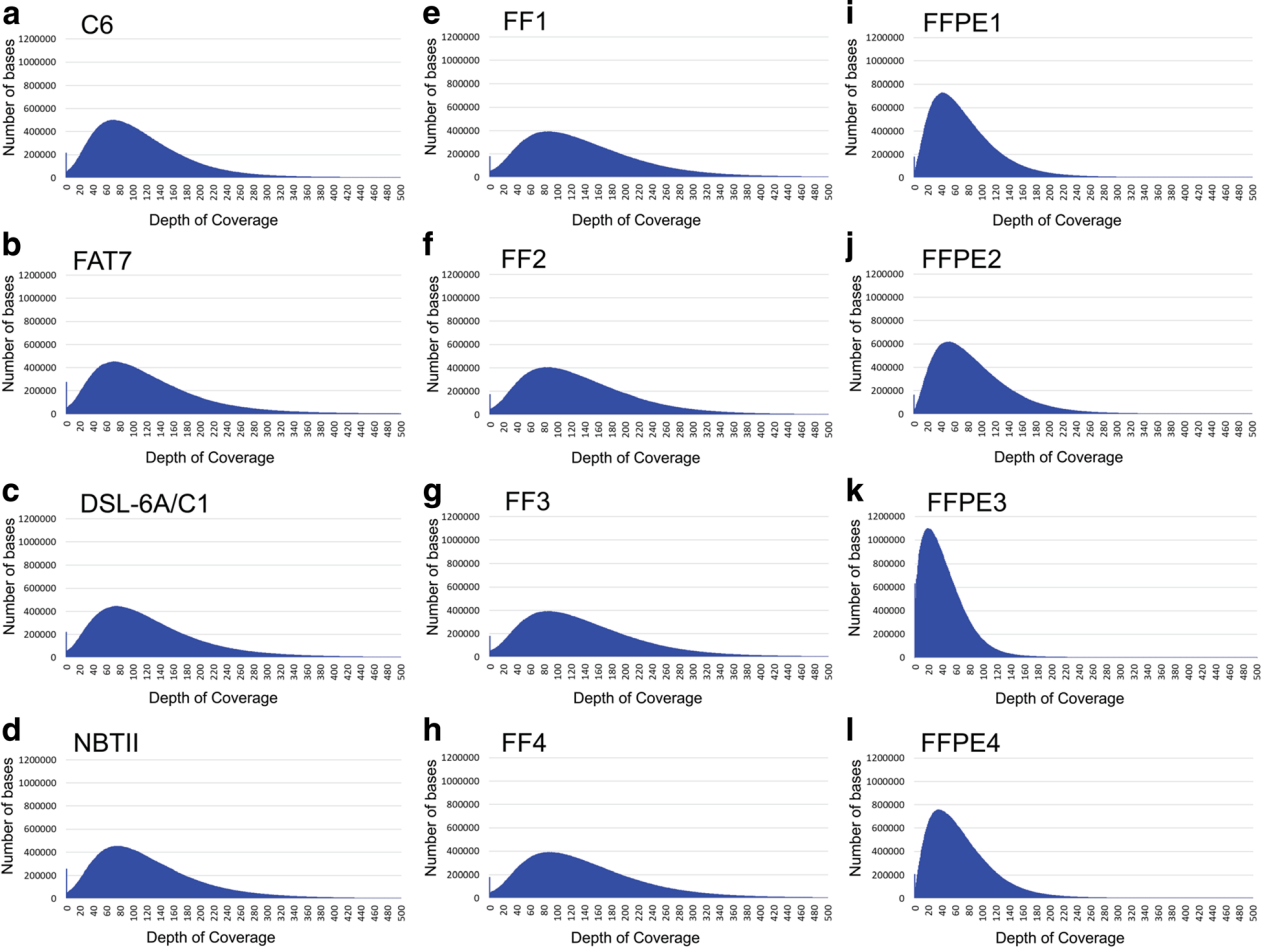
Uniformity of coverage for up to 500 bp reads for the cell lines and paired FF-FFPE samples. **a**-**d** Depth of coverage distribution for the C6, FAT7, DSL-6A/C1 and NBTII cell lines. **e**-**h** Depth of coverage distribution for the fresh frozen (FF) liver tissue. **i**-**l**) Depth of coverage distribution for the formalin-fixed, paraffin-embedded (FFPE) liver tissue

We also looked at the uniformity of coverage for up to 500 bp reads for the cell lines and paired FF-FFPE samples and found the depth of coverage consistent across samples within the cell lines, FF and FFPE groups. Cell line depth of coverage distribution peaked at ~ 80 reads per ~ 500,000 bases (Fig. 3a-d). The distribution was broader for the FF samples with the peak at about ~ 80–100 reads with 400,000 bases (Fig. 3e-h). For the FFPE samples, the peak of reads shifted to the left with decreased reads (20–40) covering 600,000–800,000 bases (Fig. 3i-l). FFPE3 was an outlier with 20–40 reads covering over a million bases, again consistent with greater DNA fragmentation. Depth of coverage plot distributions were consistent with previously published work [15].

We examined the distribution of gene reads for bias by plotting all the sample exonic variants and comparing them to the distribution of RefSeq genes across the rat chromosomes (Additional file 3: Figure S1). With the exception of chromosome 20, variant reads were uniformly distributed across all chromosomes. With regards to chromosome 20, there were several variants related to the RT1 family genes (histocompatibility complex family), perhaps indicating a data rich region accounting for the higher distribution of reads than the other chromosomes.

### Variant identification

Variant identification was a multistep process where a coverage depth of 50X and an alternate allele depth of 20X were considered discriminately stringent for sensitivity and accuracy. Variants were called using GATK v3.7 program. SNVs and INDELs were identified with the GATK v3.7 utility, HaplotypeCaller®. Exonic variants were filtered with GATK, followed by functional annotation of the variants with the SnpEff tool. Intronic and intergenic variants were removed leaving only coding regions and UTR variants (Table 3).

**Table 3:** All exonic variants detected in the rat exome-seq samples

Sample	Total Number of SNPs (%)	Annotated SNPs (%) (based on dbSNP)	Non-annotated SNPs (%) (based on dbSNP)
C6	30,529	15,945 (52.2%)	14,584 (47.8%)
FAT7	24,167	13,818 (57.2%)	10,349 (42.8%)
DSL-6A/C1	22,060	12,719 (57.7%)	9341 (42.3%)
NBTII	37,984	18,121 (47.7%	19,863 (52.3%)
FF	22,685	14,240 (62.7%)	8445 (37.2%)
FFPE	15,944	10,193 (63.9%)	5751 (36.1%)

Variant detection at 50X depth of coverage and alternate allele depth of 20x

A total of 153,369 SNPs were identified across all exonic variants in the 50 Mb exome component of the design with 85,036 registered in dbSNP (Build 149; November 7, 2016), leaving 68,333 unique SNPs. We used FF samples as the “gold standard” so that 22,685 total SNPs were identified with 14,240 annotated or registered in dbSNP and 8445 identified as non-annotated, not registered in dbSNP. Compared to the FF tissue samples, FFPE variants were substantially decreased (15,944 total, 10,193 annotated and 5751 non-annotated).

Rat tumor cell lines were selected as a proof of principle model to test the rat WES platform, since variants should be plentiful and diverse in chemically induced, tumor derived cancer cell lines. After filtering annotated and non-annotated FF-FFPE normal tissue variants from the tumor cell line data, numerous annotated and non-annotated variants based on dbSNP were identified in each cell line (Table 4).

**Table 4:** Cell line specific exonic variants detected in the rat exome-seq samples

Sample	Total Number of Variants (SNPs + INDELS)	Annotated Variants (%) (based on dbSNP)	Non-annotated Variants (%) (based on dbSNP)
C6	5387	1523 (28.3%)	3864 (71.7%)
FAT7	3551	1211 (34.1%)	2340 (65.9%)
DSL-6A/C1	3277	1069 (32.6%)	2208 (67.4%)
NBTII	10,123	2430 (24.0%)	7693 (76.0%)

Variant detection at 50X depth of coverage and alternate allele depth of 20×

NBTII, the bladder cell line contained the most variants (10,123), followed by the glioma C6 line (5387) the FAT7, formaldehyde line (3551) and the DSL-6A/C1, pancreatic cancer line (3277). To determine if there was human disease relevance between our chemically induced tumor mutations and previously identified mutations found in human malignant tumors, we matched the variants in each cell line to validated mutations present in the COSMIC database with the same amino acid substitutions and/or location as that found in our exome sequence data. We report for each tumor cell line, relevant cancer-related genes and those genes associated with a specific cancer type.

The C6 rat glioma tumor cell line, chemically induced by N,N-nitrosomethylurea in an outbred Wistar rat [18] and morphologically similar to gliobastoma multiforme, is an important human translational research model for malignant glial neoplasms [19, 20]. Exome sequencing of the C6 glioma captured 3864 non-annotated variants of which 3447 were designated as SNPs and 417 as INDELs. Genes of interest previously reported with human relevance to glioblastomas include *Cdkn2a, Cdkn2b, Tp53* and *Pik3ca*. Our results verify these reports for all these genes [20, 21]. Deletion of *Cdkn2a* and *Cdkn2b* and the absence of any *Tp53* mutations as detected by exome sequencing is in agreement with the mutant *p16/CDKn2a/Ink4a* locus [20, 21] with no expression of p16 and p19ARF mRNAs and a wildtype p53 previously reported for the C6 line [21, 22]. The oncogene, *Pick3ca*, matched the COSMIC database with an identical amino acid substitution (C90Y) and location as reported in Grade IV astrocytomas [14].

Formaldehyde, an abundantly produced chemical classified as a rodent and human carcinogen [23] has undergone extensive genotoxic, carcinogenic and teratogenic studies. Early mechanistic studies focused on *Tp53* changes, specifically a point mutation (cGt – cAt) in a well conserved region at codon 271 (R271H) [24–26]. We confirmed this point mutation using the FAT7 cell line, a formaldehyde induced squamous cell carcinoma of the nasal cavity derived from a Fisher-344 rat. This mutation was not documented in any of the other exome sequenced tumor cell lines. We identified 2340 non-annotated variants of which 1963 were designated as SNPs and 377 as INDELs. Other reported molecular endpoints characterizing formaldehyde exposure are induced missense mutations mainly at G:C base pairs [27, 28]. We checked for this mutation and found 4–5% G:C and 4–5% C:G substitutions, accounting for ~ 10% of the base substitutions in the FAT7 cell line. The majority of mutations in this line are from C:T (~ 20%) and G:A (~ 20%). It is important to note these results are not specific to the formaldehyde-exposed cell line, as they are seen in each of the evaluated cell lines. Exome sequencing can help distinguish true molecular changes secondary to formaldehyde exposure and those specific to squamous cell carcinomas lending additional insight into mechanisms of formaldehyde-induced carcinoma, which could hold significant relevance for environmental or occupational exposure studies.

DSL-6A/C1 is a pancreatic ductal carcinoma cell line derived from a transplantable DSL-6 acinar cell carcinoma of an azaserine-treated Lewis rat [29]. Exome sequencing identified 2208 non-annotated variants, of which 1815 were SNPs and 393 were INDELs. A mutation unique to DSL-6A/C1 is *Ctnnb1*, a reported gene in 20–25% of human acinar cell carcinomas [30]. Our exome-seq data for this mutation matched the COSMIC database location and amino acid substitution. As in the C6 glioma line, there were homozygous deletions of *Cdkn2a* and neighboring *Cdkn2b*. This alteration reportedly occurs more frequently in human pancreatic ductal carcinomas [31]. *Tp53* lacked any genetic alterations. Rat exome-seq findings for DSL-6A/C1 were concordant with human pancreatic acinar cell carcinoma genetic alterations. Acinar cell carcinomas lack frequent alterations in genes commonly mutated in pancreatic ductal adenocarcinoma such as *KRAS* and *Tp53* [30, 31]. Although the ATCC specifies the DSL-6A/C1 line as a pancreatic ductal cell carcinoma from a transplanted acinar cell carcinoma, the ductal phenotype is not adopted until after 2–3 weeks of cultured growth when the cells lose amylase production [29]. Since we processed the cells directly after thawing and did not culture, it appears the acinar cells possess both acinar (*Cttnb1*, wild type *Tp53*) and ductal (deletion of *Cdkn2a* and *Cdkn2b*) traits related to each pathologic phenotype.

The total number of variants (10,123) in the NBTII bladder carcinoma cell line induced in a Wistar rat treated with N-butyl-N-(4-hydroxybutyl)nitrosamine far exceeded the total number of variants called in the other three cell lines (Table 4). Non-annotated SNPs (7693), and INDELs (828) were identified. Two *Tp53* point mutations present in codons 211 (R211W) and 230 (I230T) had the same amino acid substitutions and location found in the COSMIC database. Missense mutations were recognized in *Ncor1* and *Pik3ca*, oncogenes of central importance to human cancer as known driver mutations. Other exonic variants identified by the rat WES platform with relevance in human bladder carcinoma include *RB1, Muc16, Muc5b, NAT1, NAT2, Stag2, Stag3* and *Arid1a*. All these genes were unique to the NBTII bladder cell line.

### Sequence validation

Sanger sequencing provided an orthogonal means of testing data accuracy. We analyzed a select set of called variants of moderate (non-synonymous, missense, nonsense) to high (frameshift muations and INDELS) impact in cancer-related genes with exonic variants unique to a single cell line or found across multiple lines. Furthermore, to add richness to our validation assessment, we included cancer genes with mutations matching amino acid substitutions and/or locations in the COSMIC database. The first gene validation set consisted of highly relevant “common” cancer genes (*Tp53, Ncor1, Pik3ca, Cdkn2a* and *Cdkn2b*) shared by many tumors. A second set consisted of an additional 15 cancer-related genes (*Nf1, Mki67, Mllt4, Fgfr2, Ctnnb1, Fat1, Clp1, Fat4, Arid1a, Nat1, Nat2, Setd2, Impg1, Nbas* and *Npat*) (Table 5). We confirmed all exome-seq variants in the “common” gene set in all four tumor cell lines; the presence of *Tp53* mutations in the FAT7 (R271H) and NBTII lines (R211W, I230T), and its absence in the C6 and DSL-6A/C1 cell lines, missense mutations for the oncogenes *Ncor1* (E544D) across all four cells lines and *Pick3ca* in the C6 (C90Y) and the NBTII (M811 T) cell lines. All mutations in the ‘common’ gene set matched the variant calls in the human COSMIC database. Homozygous deletions of *Cdkn2a* and *Ckdn2b* were confirmed by qPCR analysis in cell lines C6 and DSL-6A/C. There was complete concordance between the rat WES and Sanger sequencing methods for the common gene set.

**Table 5:** Variant candidate validation by Sanger sequencing

Chromosome #: Position	Gene	Exon	Codon Change	C6			FAT7			DSL 6A/C1			NBTII		
				Coverage		AF	Coverage		AF	Coverage		AF	Coverage		AF
				Total	Allele		Total	Allele		Total	Allele		Total	Allele	
Chr10:56196111	***Tp53*** ^a^	R271H	cGt/cAt	WT	WT	–	**71** ^**d**^	**71**	**1.000**	WT	WT	–	WT	WT	–
Chr10:56195619	***Tp53***	R211W	Cgg/Tgg	WT	WT	–	WT	WT	–	WT	WT	–	*197* ^**e**^	*113*	*0.574*
Chr10:56195677	***Tp53***	I230T	aTc/aCc	WT	WT	–	WT	WT	–	WT	WT	–	*174*	*90*	*0.529*
Chr10:48699815	***Ncor1***	E544D	gaG/gaC	**194**	**85**	**0.438**	**157**	**157**	**1.000**	**166**	**166**	**1.000**	**191**	**191**	**1.000**
Chr2:118851700	*Pik3ca* ^b^	M811 T	aTg/aCg	WT	WT	–	WT	WT	–	WT	WT	–	*227*	*111*	*0.489*
Chr2:118831618	***Pik3ca***	C90Y	tGt/tAt	*129*	*62*	*0.481*	WT	WT	–	WT	WT	–	WT	WT	–
Chr5	Cdkn2a	Deletion		WT	WT	–	WT	WT	–	WT	WT	–	WT	WT	–
Chr5	Cdkn2b	Deletion		WT	WT	–	WT	WT	–	WT	WT	–	WT	WT	–
Chr10:66790063	Nf1	Q962*	Cag/Tag	*151*	*79*	*0.523*	WT	WT	–	WT	WT	–	WT	WT	–
Chr1:208011800	Mki67	G207R	Ggg/Agg	***314/−*** ^***f***^	***68/−***	***0.217/−***	WT	WT	–	***442/−***	***202/−***	***0.457/−***	WT	WT	–
Chr1:53731379	Mllt4	Q1440*	Caa/Taa	WT	WT	–	***111/−***	***30/−***	***0.270/−***	WT	WT	–	WT	WT	–
Chr1:200672370	Fgfr2	V70 M	Gtg/Atg	**126**	**126**	**1.000**	**159**	**159**	**1.000**	WT	WT	–	WT	WT	–
Chr8:129618253	***Ctnnb1***	D32V	gAt/gTt	WT	WT	–	WT	WT	–	*55*	*20*	*0.364*	WT	WT	–
Chr16:50398579	Fat1	L2464P	cTc/cCc	WT	WT	–	WT	WT	–	WT	WT	–	*154*	*62*	*0.403*
Chr16:50485429	Fat1	M536 T	aTg/aCg	*131*	*67*	*0.511*	*149*	*149*	*1.000*	WT	WT	–	*196*	*98*	*0.500*
Chr16:50485954	Fat1	F361S	tTc/tCc	WT	WT	–	**175**	**175**	**1.000**	WT	WT	–	WT	WT	–
Chr3:72125722	***Clp1*** ^c^	M417	atg/	WT	WT	–	WT	WT	–	***165/−***	***25/−***	***0.152/−***	WT	WT	–
Chr2:125846677	***Fat4***	I3077V	Att/Gtt	WT	WT	–	WT	WT	–	**200**	**200**	**1.000**	WT	WT	–
Chr2:125754029	***Fat4***	S627 T	aGt/aCt	WT	WT	–	WT	WT	–	WT	WT	–	*94*	*37*	*0.394*
Chr2:125754184	***Fat4***	L679F	Ctc/Tcc	WT	WT	–	WT	WT	–	WT	WT	–	*143*	*84*	*0.587*
Chr5:151918885	***Arid1a***	Y658*	taT/taA	WT	WT	–	WT	WT	–	WT	WT	–	*78*	*29*	*0.372*
Chr16:23972323	Nat1	S15 L	tCa/tTa	WT	WT	–	WT	WT	–	WT	WT	–	*300*	*154*	*0.513*
Chr16:23961976	***Nat2***	L52*	tTa/tAa	WT	WT	–	WT	WT	–	WT	WT	–	*197*	*90*	*0.457*
Chr8:118824049	***Setd2***	R792TEPSVR	agg/aCTGAACCTTCAGTTAgg	WT	WT	–	**95**	**95**	**1.000**	**103**	**103**	**1.000**	WT	WT	–
Chr8:118824049	***Setd2***	R79TESSVR	agg/aCTGAATCTTCAGTTAgg	WT	WT	–	WT	WT	–	WT	WT	–	120	38	0.317
Chr8:87845140	***Impg1***	T133I	aCc/aTc	*151*	*74*	*0.490*	WT	WT	–	WT	WT	–	WT	WT	–
Chr6:38567303	***Nbas***	T837 N	aCc/aAc	56	56	1.000	***WT/+***	***WT/+***	–	60	60	1.000	***WT/+***	***WT/+***	–
Chr6:38567315	***Nbas***	A841V	gCg/gTg	53	53	1.000	***WT/+***	***WT/+***	–	58	58	1.000	***WT/+***	***WT/+***	–
Chr8:58154526	***Npat***	K1186R	aAg/aGg	68	68	1.000	50	50	1.000	WT	WT	–	WT	WT	–

^a^In the gene column, bold and italics indicates the variant found in the rat exome platform matches the amino acid substitution and location in COSMIC

^b^In the gene column, italics indicates the variant found in the rat exome platform matches only the amino acid substitution in COSMIC

^c^In the gene column, bold, italics and underline indicates the variant found in the rat exome platform matches only the location in COSMIC

^d^In a cell line column, bold cell line indicates a homozygous mutation

^e^In a cell line column, italicized indicates a heterozygous mutation

^f^In a cell line column, Rat WES platform variant call/Sanger-based sequencing variant call. (WT: -, Variant: +). Bold and italics implies a sequencing discrepancy between the WES and Sanger

Next, we confirmed genotypes from cancer-related genes assigned to the second validation set. We identified 38 variants in 15 genes of which only four were inaccurately called. These miscalls were possibly due to low coverage or low allelic frequency. Associated with 12 of the genes were 26 variants where the amino acid substitution and/or location matched the validated mutations in COSMIC database. False negative calls (× 4) found by Sanger were identified in a single gene, *Nbas*. The variant calls (T837 N and A841V) were correctly called in the C6 and DSL-6A/C1 tumors lines; however, these mutations were missed by WES in the FAT7 and NBTII lines.

### Mutation Spectrum

**Fig. 4:**
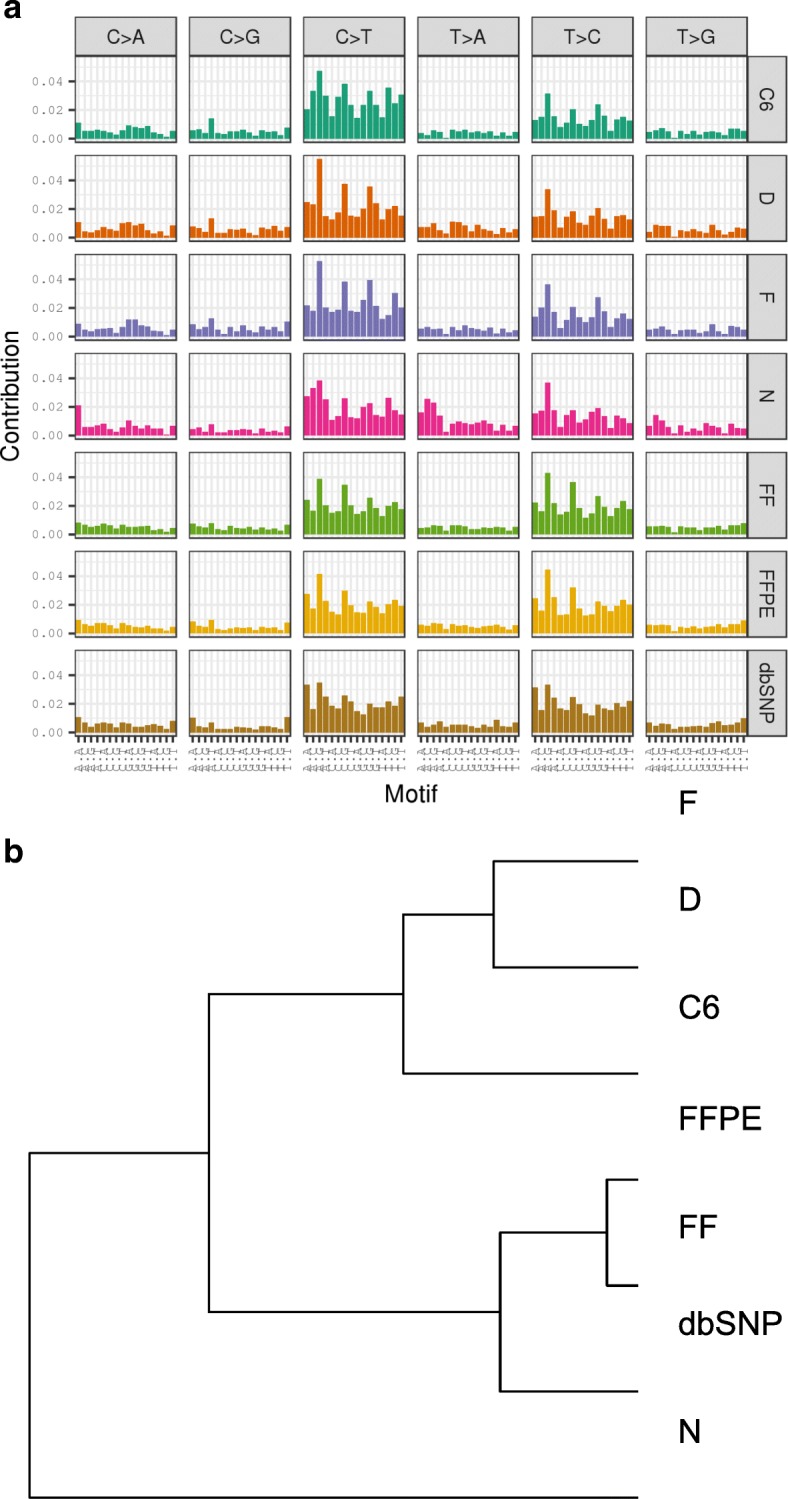
Mutational spectrum of the rat exome-seq data. **a** All exonic variants captured across all samples in the 71 Mb design (50 Mb + UTRs) plus the dbSNP variants were plotted using the Kullback-Leibler divergence. **a** A high frequency of C > T and T > C mutations presented with minimal observed differences in the mutational spectrum across all samples and dbSNP. **b** Hierarchical clustering grouped dbSNP with the normal, FF-FFPE tissue and showed divergence of these groups from the tumor cell lines

**Fig. 5:**
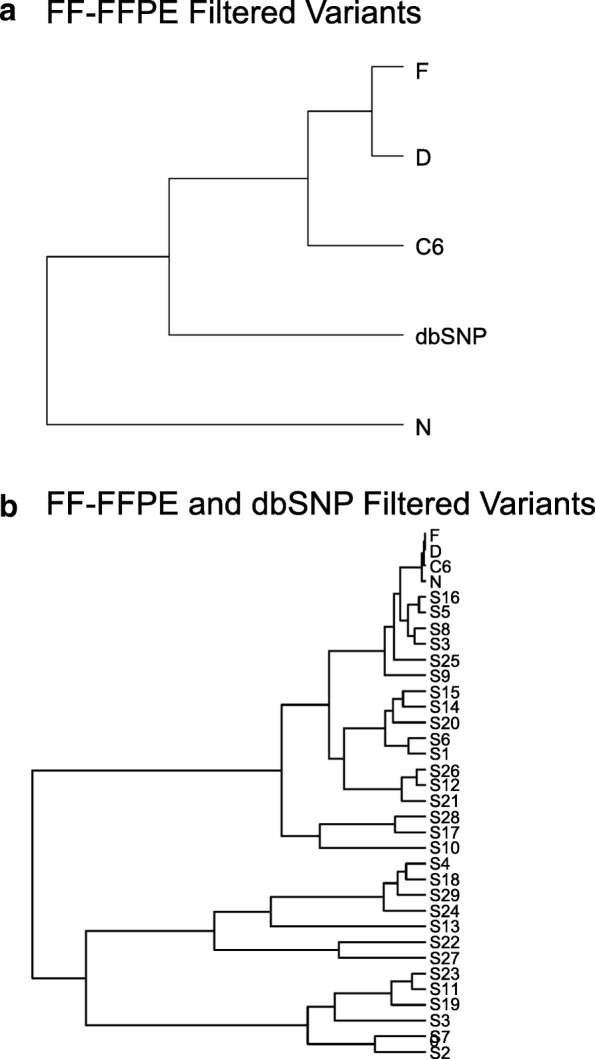
Mutational spectrum of cell line specific exonic variants. **a** Filtered for FF-FFPE variants from the exome-seq data separated dbSNP from the tumor cell line samples. **b** Based on the hierarchical clustering of cell specific variants filtered from FF-FFPE and dbSNP variants, the mutational spectrum of the rat exome-seq data is closest to COSMIC Signatures 16, 5, 8 and 3

We looked at all exonic variants with respect to the rat reference genome, Rn6, and analyzed the mutational spectrum using the Kullback-Leibler divergence to compare the mutation spectrum across each sample type [32]. Based on the mutation spectrum for each of the respective samples, we then focused on discriminating variants unique to each cell line and FF-FFPE samples and explored if there was a COSMIC signature relevant to a respective cell line indicating a high frequency of base pair mutations matching a specific tumor type. For the first comparison, we plotted dbSNP-registered variants along with all the exonic variants in the cell line and the FF-FFPE groups and observed similar frequency patterns across all samples with a very high frequency of C > T and T > C substitutions in the rat exome-seq data (Fig. 4a). Analysis of the mutation spectrum using the Kullback-Leibler method clustered dbSNP with the normal, FF-FFPE liver tissue and showed divergence of these groups from the tumor cell lines (Fig. 4b). We believe the bladder cell line, NBTII, clustered by itself due to the large number of respective variants identified with this cell line. Divergence plots focusing on the discriminating variants unique to each cell line, with the FF-FFPE variants filtered out, resulted in separation of the cell lines from dbSNP (Fig. 5a). Hierarchical clustering of the unique cell line specific exonic rat variants with the COSMIC signatures clustered the tumor cell line samples with the COSMIC signatures 16, 5, 8 and 3 (Fig. 5b). While COSMIC Signature 16 correlates to liver cancer and Signature 5 to all cancer types, both exhibit transcriptional strand bias for T > C mutations. Signature 8 associates with medullobastomas, possibly linking an association with the chemically induced C6, glioblastoma cell line. Signature 3 aligned its pancreatic cancer phenotype with the chemically induced, pancreatic tumor cell line, DSL-6A/C1. The data lends support to a possible relationship between the chemically induced, tumor cell line variants and human COSMIC mutational signatures relative to a specific cancer phenotype.

## Discussion

High throughput molecular-based methods like whole exome sequencing are needed to explore experimental in vitro systems and animal models for mechanisms and affected pathways in toxicity and disease. Yoshihara et al. recently published a probe design and application of a target capture sequencing of rat exons and conserved non-coding regions of 13 vertebrates [8]. Like the TargetEc probe design developed by Yoshihara, we employed merged RefSeq and Ensembl gene annotations to cover a 50 Mb exome region. However, in the final target capture design, Yoshihara added highly conserved, non-coding regulatory regions which tend to be shorter than exonic regions at about 5 bp in length. To compensate for the shorter regions (~ 5 bp), the probes were extended 100 bp to ensure efficient target region capture, which significantly increased the total target capture size to 146.8 Mb. The larger probe library size may be viewed as a drawback since more reads would be required to obtain comparable sequencing depth for mutation detection in genomic regions of interest [8]. The current rat exome probe set at ~ 71 Mb in size focused on sequencing the protein coding regions plus UTRs. At half the size of the TargetEC platform, the probe set presents a more feasible approach to WES analysis in the rat.

The validity of exome sequencing relies on its ability to capture accurately targeted regions of interests. In evaluating the performance of a HTS platform, specific metrics looking at the target capture, alignment to the reference genome, variant calls and validation were taken into account. All performance metrics scored favorably with high capture sensitivity and specificity for the described rat exome platform design. This newly developed tool for rat exome sequencing makes feasible a comprehensive analysis of exonic variants across the entire rat exome and increases the possibilities for the discovery of gene mutations compared to candidate gene approaches. As expected, a diverse set of highly relevant, cancer-related genes were identified in the four rat tumor cell lines. Rat exome-sequencing targeted and captured gene mutations unique to each cell line and tumor type with a number of these genes previously reported in the literature for the respective human cancer type emphasizing the importance of rat cell lines in human translational research.

One of the first steps in analyzing sequencing data for probable candidate mutations is filtering the data for annotated, registered SNPs in dbSNP. The database reports far fewer SNPs for the rat compared to the mouse and human, emphasizing the point, that the rat genome is not currently as well annotated as its lab counterpart, the mouse. Our data set confirmed in all samples, the detection of annotated variants based on dbSNPs as well as the discovery of several non-annotated SNPs. Additional exome sequencing with this rat exome capture method can add valuable genomic data to rat databases.

Fresh frozen tissue is the “gold standard”, sample type of choice, for high throughput sequencing studies widely used to characterize variations from both normal and diseased tissues. Since these tissues do not undergo formalin fixation and processing at high temperatures, the DNA integrity is not altered. Based on capture sensitivity and specificity, the findings from this study show FFPE sample performance on the rat WES platform were comparable to FF tissue. Even though differences in the capture sensitivity and specificity existed between the two groups, the differences were marginal. At a coverage depth of 50X, our results affirmed FFPE tissues with a mean capture specificity of 57%. Capture specificity for FFPE samples evaluated at less stringent, but still strong coverage at an average sequencing depth of 30X resulted in an improved reference genome coverage to ~ 77%. As processed under conditions described herein, FFPE samples were within the limits of achieving sequencing results comparable to FF tissue. Numerous clinical studies compared FF and FFPE specimens from normal tissue and from various tumor types and achieved concordant, reliable sequencing results [33–35]. Although, caveats of nucleic acid degradation and protein crosslinking have been well described for FFPE tissue [34, 36], multiple sequencing studies have successfully demonstrated sufficient capture of target regions with acceptable capture sensitivity and specificity to obtain reliable sequencing data. We confirm the utility of using FFPE samples for exome-seq analysis in the current study.

COSMIC (Catalogue of Somatic Mutations in Cancer) is a public cancer database of human somatic mutations with data curated from the scientific literature and large-scale genomic studies from the Cancer Genome Project at the Sanger Institute [14]. Since the rat and its cell lines are widely used in human translational disease studies, we matched variants identified in cancer-related genes found in our tumor cell line samples to reported mutations in these same genes in COSMIC. We selected a set of genes (*Tp53*, *Ncor1* and *Pik3ca*) common to multiple cancer types and evaluated the condcordance of the captured exonic sequence by Sanger-based sequencing. The probe set we designed matched mutations with the identical amino acid substitutions found in the COSMIC database. In addition, qPCR analysis confirmed homozygous deletions of *Cdkn2a* and *Cdkn2b*, another common deletion present in multiple human cancers. The successful validation and matching of driver mutations found in common cancer genes suggests the rat WES probe set described here should be an immensely effective tool and impact human cancer genomic translational studies.

Sanger dideoxy terminator sequencing is the widely accepted “gold standard” for validating variants found using NGS even with its limitations as an orthogonal test [37]. We performed extensive confirmation of variants identified by WES to confirm the platform’s performance and ability to capture a wide-spectrum of mutations. Compared to other experimental animal models for which a probe set was designed to capture the exome, we assessed variant concordance between our rat exome method and Sanger sequencing on a sizeable set of variants to assure the accuracy of our in-silico probe design approach [8, 38, 39]. Our validation rate compared very favorably with Sanger data and represents a high degree of accuracy for the rat exome capture and sequencing platform.

When conducting experimental animal studies, accounting for strain differences within a species is an important aspect of the study design. In spite of the strain divergence among the cell line and tissue samples in our study, the total number of reads mapped well to the reference genome of Rn6, which is from the BN/SsNHsdMCW (Brown-Norway) strain. The percentage of aligned reads for the cell line and fresh frozen samples was 99%. The FFPE alignment was 90%. Although the probe sequence design was based on the Brown-Norway reference strain, and cell line variant comparisons were not matched to the respective rat strain, our designed probe set worked successfully for inbred and outbred laboratory rat strains for the identification of cancer-related genes, consistent with COSMIC annotation.

Human oncology studies employing genomic or exome sequencing methods often compare mutational profiles to defined cancer-associated mutational signatures in the COSMIC database. This allows insight into somatic mutations shared by a population and mechanisms driving cancer. Using tissue, kidney cell lines and embryonic stem cell lines from *Fhit* knockout mice, Volinia et al. compared the mutation profile to signatures in the COSMIC database and established that a loss of gene expression controlled development of the ubiquitous signature 5 mutations in human cancer [40]. This discovery was based on the exome sequencing of the mouse, a highly annotated experimental animal model. For the current study, hierarchical clustering of four rat tumor cell lines coincided with defined molecular and cancer tissue signatures in the COSMIC database. Further work will continue to improve annotation of the rat genome and better establish the relationship between chemically induced rat tumors and defined human cancer-associated signatures in the COSMIC database.

## Conclusions

A whole exome capture probe set and NGS sequencing of the rat exome represents a whole genome sequencing approach for investigators working in drug discovery, toxicology and hazard assessment. Targeted exome enrichment and sequencing can efficiently lead to variant discovery for insights into toxicity or disease etiologies, such as non-neoplastic and neoplastic lesions, along with developmental, reproductive, neurological, metabolic and endocrine disorders. The rat is an important experimental animal model used extensively in academia, industry and governmental agencies for toxicity and carcinogenicity assessment of various drugs, chemicals and hazardous agents. This newly developed rat exome enrichment system will expand the NGS tools available for rat genomic research.

## Additional files

Additional file 1: **Table S1.** Rat exome-seq 500 flanking bp primer design for validation. (XLSX 20 kb)

Additional file 2: **Table S2.** PCR forward and reverse primer pairs for Sanger sequencing. (XLSX 10 kb)

Additional file 3: **Figure S1.** Exonic Variant Read Distribution. The distribution of all exonic variant reads across all chromosomes is directly proportional to the number of RefSeq genes for each chromosome, except chromosome 20. (PDF 15 kb)

## Abbreviations

COSMIC: Catalogue of Somatic Mutations in Cancer
D: DSL-6 A/C1
dbSNP: Single Nucleotide Polymorphism database
F: FAT7
FF: Fresh frozen
FFPE: Formalin-fixed paraffin-embedded
HTS: High throughput screening
INDEL: Insertion/deletion
M: Million
N: NBTII
NGS: Next generation sequencing
SNV: Single nucleotide variant
UTR: Untranslated regions
WES: Whole exome sequencing
WT: Wild type
