# Supplementary figures and images for: Whole exome sequencing in the rat
Source: BMC Genomics. 2018 Jun 20;19:487. doi: 10.1186/s12864-018-4858-8 (PMC6011395; doi:10.1186/s12864-018-4858-8)

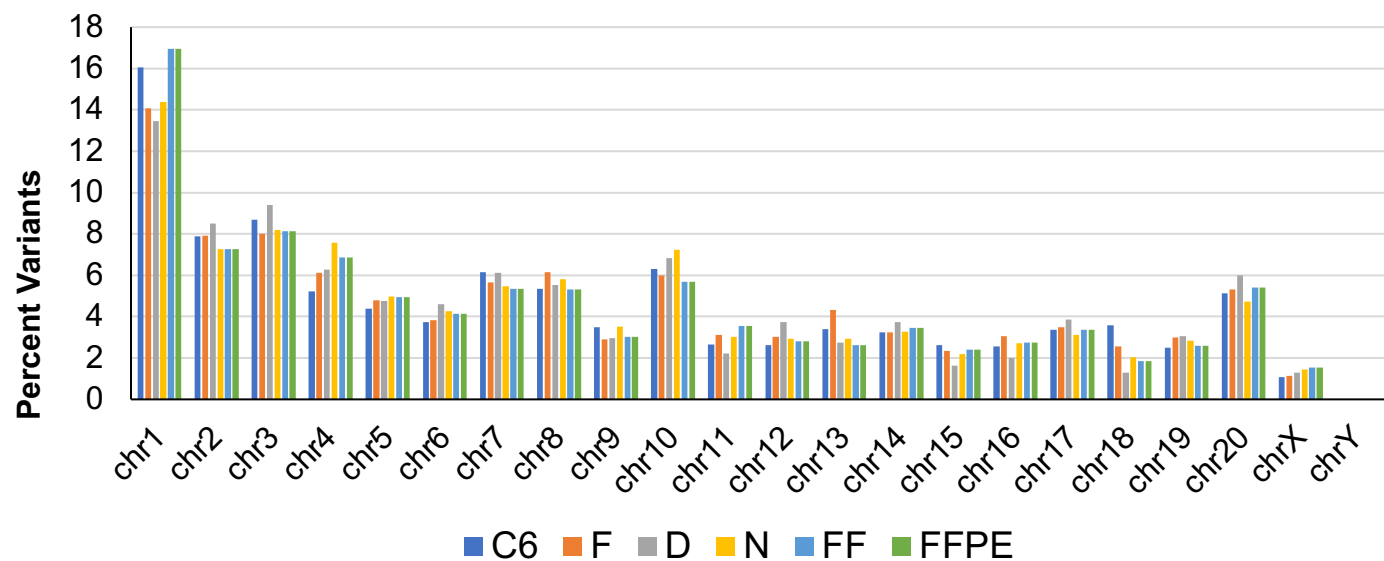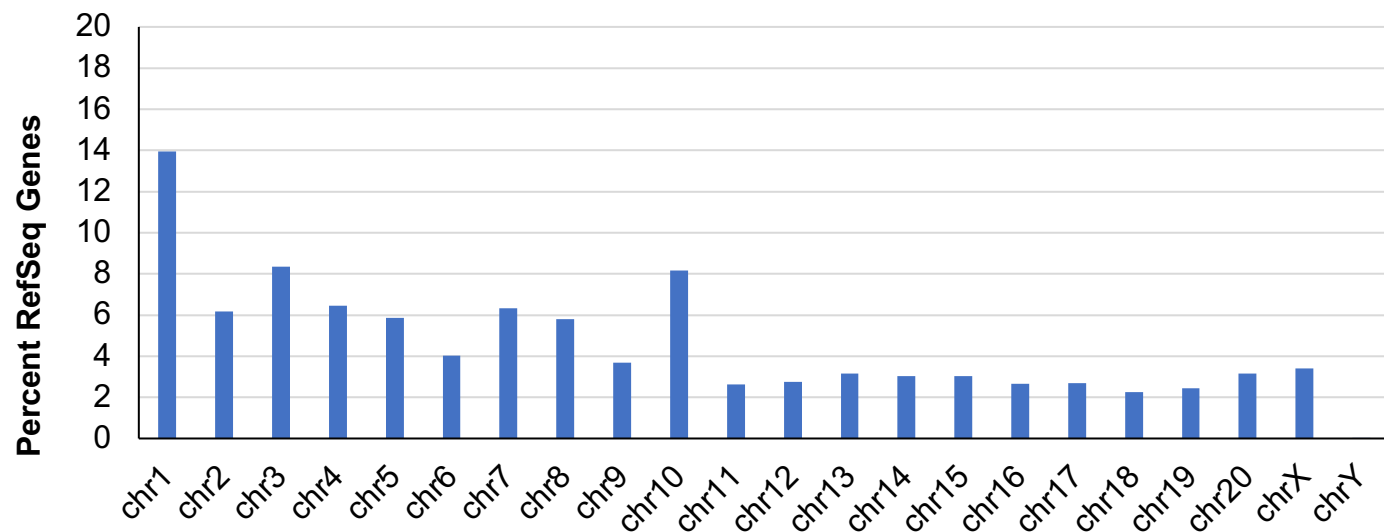

Supplement: Supplementary file 3 — Figure S1. Exonic Variant Read Distribution. The distribution of all exonic variant reads across all chromosomes is directly proportional to the number of RefSeq genes for each chromosome, except chromosome 20. (PDF 15 kb) [file 12864_2018_4858_MOESM3_ESM.pdf]
